# Improvement in the Image Quality of Bile and Pancreatic Ducts with Dual-Layer Spectral CT

**DOI:** 10.2174/0115734056442763260317072250

**Published:** 2026-04-08

**Authors:** Xiaoyi Bai, Kai Liao, Zhihan Wu, Mo Chen, Wang Rui, Kai Deng

**Affiliations:** 1 Department of Gastroenterology and Hepatology, West China Hospital, Sichuan University, Chengdu, China; 2 Department of Gastroenterology & Hepatology, Sichuan University-University of Oxford Huaxi Joint Center for Gastrointestinal Cancer, West China Hospital, Sichuan University, Chengdu, China; 3 Department of Radiology, West China Hospital, Sichuan University, Chengdu, China; 4 Department of General Practice, Hospital of Chengdu Office of People’s Government of Tibetan, Chengdu, China; 5 Department of General Practice, Tibetan Chengdu Branch Hospital of West China Hospital, Sichuan University, Chengdu, China

**Keywords:** Dual-layer spectral detector CT, Virtual monochromatic images, Biliary duct imaging, Pancreatic duct imaging, Image quality optimization, Radiomic feature stability

## Abstract

**Background:**

While multiple imaging modalities have been developed for the evaluation of biliary and pancreatic ducts, none is considered ideal. Dual-layer Spectral Computed Tomography (DSCT) offers potential advantages through dual-energy acquisition and spectral imaging, but its application for biliopancreatic ducts remains unclear. The objective of this retrospective observational study was to evaluate image quality and determine the optimal energies of Virtual Monoenergetic Images (VMIs) from DSCT of biliary and pancreatic ducts.

**Materials and Methods:**

We analyzed contrast-enhanced abdominal DSCT images of 75 patients with normal ductal anatomy. Both conventional Polyenergetic Images (PEIs) and VMIs at energy levels ranging from 40–140 keV (10-keV intervals) were reconstructed. The ductal system was categorized into four groups: intrahepatic bile ducts (Group A), extrahepatic bile ducts (Group B), pancreatic ducts (Group C), and the main pancreatic ducts (Group D). Objective image quality parameters, including CT values, image noise, the Contrast-to-Noise Ratio (CNR), and the Signal-to-Noise Ratio (SNR), were systematically measured and compared across different energy levels.

**Results:**

Compared with the PEIs, the VMIs reconstructed at low keV levels *via* DSCT demonstrated superior image quality across the four groups. Significant differences in CNR values were observed between VMIs at 40 keV and PEIs in Group A [4.30 (2.79, 6.15) *vs.* 3.37 (2.52, 4.32), *p* = 0.013], Group B [10.46 (8.41, 12.43) *vs.* 5.83 (4.47, 6.94), *p* < 0.001], Group C [11.50 (9.05, 14.48) *vs.* 5.34 (4.31, 6.56), *p* < 0.001], and Group D [9.11 (7.00, 11.86) *vs.* 5.36 (4.34, 6.95), *p* < 0.001].

**Discussion:**

This suggests a clear advantage of low-energy VMIs in depicting subtle ductal anatomy that is poorly visualized on standard CT. The consistent reduction in image quality metrics with increasing energy further supports the use of low-keV settings for soft-tissue duct evaluation. Limitations, including the retrospective design and single-vendor platform, are acknowledged.

**Conclusion:**

Compared with conventional images, VMIs at low keV exhibit superior image quality; thus, VMIs may be incorporated into routine clinical imaging protocols.

## 1 INTRODUCTION

Biliary and pancreatic duct diseases often evade early detection and present at advanced stages, which leads to profound psychosocial and healthcare system impacts. The key to early and accurate diagnosis for effective treatment lies in the clear visualization of internal lesions within these ducts, a task that currently relies heavily on imaging examinations. Although various imaging modalities, including Ultrasound (US) with its limited sensitivity, Magnetic Resonance Cholangiopancreatography (MRCP) with its lower spatial/temporal resolution and high cost, and Endoscopic Retrograde Cholangiopancreatography (ERCP), which is invasive, are available, none is considered ideal. While Computed Tomography (CT) provides high spatial resolution for biliary and pancreatic duct imaging, its intrinsically low soft-tissue contrast fundamentally limits clear delineation of the ductal anatomy [1]. Studies have demonstrated significantly lower detection rates for pancreatic cancer and choledocholithiasis with CT than with Magnetic Resonance Imaging (MRI), particularly when the absence of bile duct dilatation markedly reduces the sensitivity of CT for stone detection [2]. These limitations underscore the need for a more precise, convenient, and safe imaging modality that can reliably visualize subtle ductal structures, which is critical for increasing diagnostic accuracy for pancreaticobiliary diseases.

In Dual-layer Spectral Detector Computed Tomography (DSCT), two detector layers and an X-ray tube are used to simultaneously collect low- and high-energy data without the need for additional steps or contrast media. Numerous studies have validated its clinical application in gastrointestinal oncology (including colorectal and gastric carcinoma imaging for tumor characterization, histological classification, and staging) [3-6], and vascular imaging (including cerebrovascular and coronary artery evaluation) [7, 8]. In addition to conventional Polyenergetic Images (PEIs), projection space decomposition is employed to generate spectral basis images in DSCT. This enables the generation of multiple spectral images, including material decomposition images (*e.g.*, iodine-only, virtual noncontrast, and effective atomic number maps) and Virtual Monoenergetic Images (VMIs). These images increase vascular contrast, improve lesion visibility, reduce artifacts, and decrease radiation dose [9]. Additionally, changes in the energy levels of VMIs can affect the repeatability and stability of radiomic features [10]. An optimal energy level may increase the discriminatory capacity of different tissues [11, 12]. However, data on the impact of low-energy VMIs on biliary-pancreatic system imaging remain scarce.

Therefore, the purpose of this study was to evaluate image quality and determine the optimal energies of VMIs constructed from DSCT, ranging from 40 to 140 keV for biliary and pancreatic duct imaging, compared with those of PEIs. Since significant biliary or pancreatic duct dilation caused by overt diseases can increase the difficulty of detection by conventional CT, potentially obscuring the relative advantages of DSCT, patients with normal ductal anatomy were enrolled in the present study. This approach allows a more focused investigation into the potential improvements in differentiation capability offered by DSCT.

## 2 MATERIALS AND METHODS

### 2.1 Patients

We included patients who underwent intravenous contrast-enhanced abdominal DSCT at our hospital from October 2023 to April 2024. The exclusion criteria were as follows: (1) incomplete imaging of the biliary and pancreatic ducts; (2) underlying pathologies known to cause pathological dilation of the pancreaticobiliary ductal system (*e.g.*, choledocholithiasis, pancreatic carcinoma, cholangiocarcinoma, chronic pancrea-titis, or ampullary tumors); (3) severe artifacts on CT images; and (4) incomplete clinical data.

### 2.2 CT Examination Protocol

A CT examination was performed using a Spectral CT 7500 scanner (Philips Healthcare, Best, The Netherlands). The helical scan range extended from the diaphragm to the pubic symphysis. Initially, a traditional CT scan was performed at 120 kVp. Patients subsequently received an intravenous injection of 70–100 mL of nonionic iodinated contrast material (Iopamidol 370 mg/mL; Shanghai Bracco Sine Pharmaceutical Co., Ltd., China) at a rate of 2.5–3 mL/s *via* a power injector, which corresponds to 1.0–1.2 mL per kilogram of body weight. Contrast-enhanced CT scans of the abdomen were then conducted during the venous phase *via* spectral CT imaging. Bolus tracking was employed, with the aorta as the region of interest. Image acquisition began 7 seconds after the signal attenuation reached the predefined threshold of 100 Hounsfield Units (HUs) for the arterial phase. The venous phase utilized a 30-second delay. The scanning parameters were as follows: tube current, automated exposure modulation; detection collimation, 128 × 0.625 mm; rotation time, 0.4 seconds; and helical pitch, 1.1. All axial section images were reconstructed at a slice thickness and interval of 2 mm. The PEIs were reconstructed *via* the iDose^4^ algorithm, whereas the spectral images were generated *via* spectral reconstruction at level 4 [13].

### 2.3 Image Generation

The PEIs were obtained, and the quantitative spectral analysis was performed using IntelliSpace Portal software (Version 12.0; Philips Healthcare). The spectral-based image data were postprocessed to generate VMIs ranging from 40 to 140 keV with 10 keV intervals. Only the images generated from the arterial phase were evaluated.

### 2.4 Objective Image Analysis

All the images were annotated by the same researcher, who was familiar with abdominal radiology and blinded to the clinical and pathological information of the patients.

**Fig. (1):**
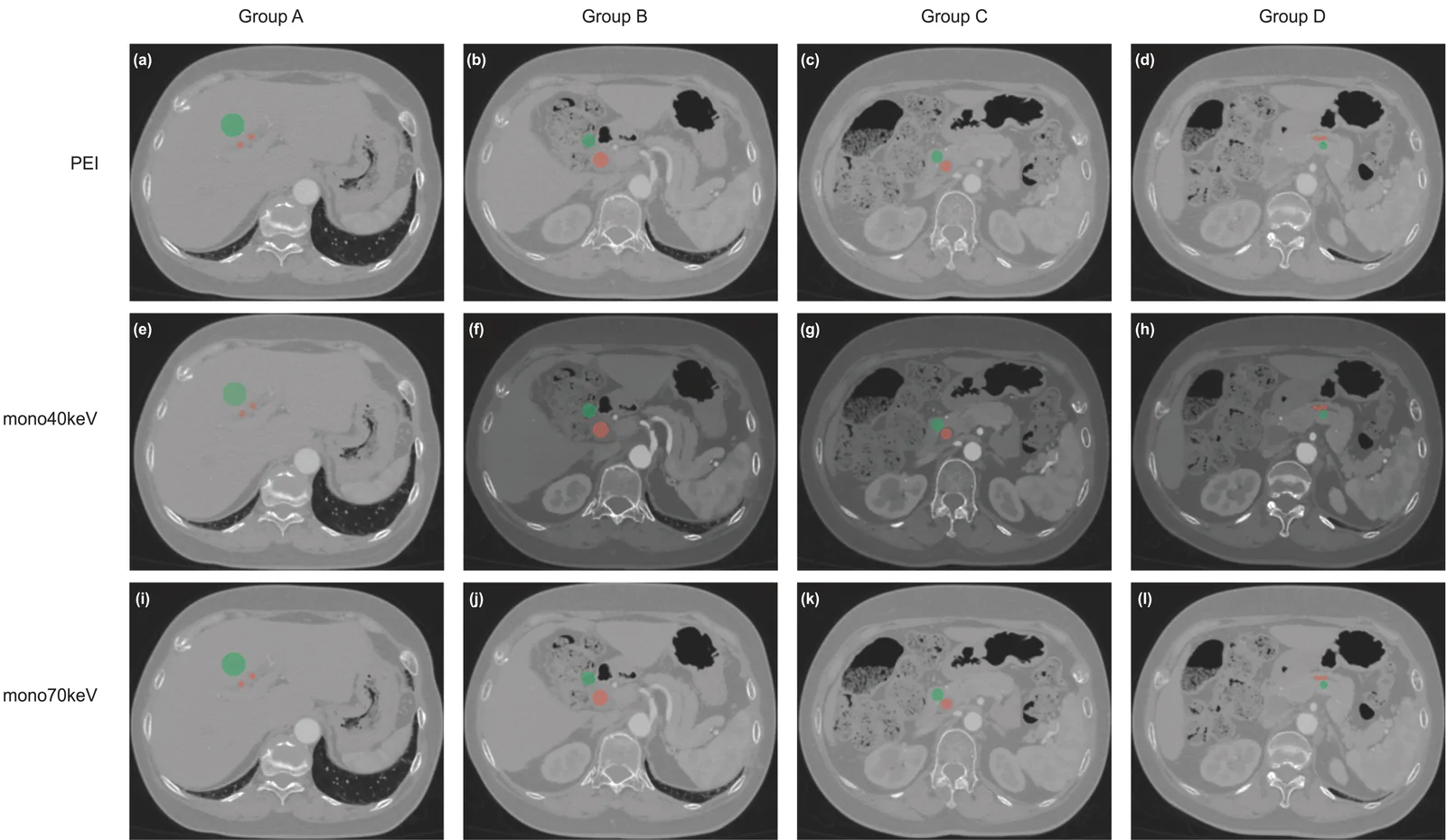
Spectral CT images of representative PEIs from the four groups at 40 keV and 70 keV. Red represents the target, and green represents the reference. PEIs (**a**–**d**), 40 keV (**e**–**h**) and 70 keV (**i**–**l**). Group A: Intrahepatic bile ducts; Group B: Extrahepatic bile ducts; Group C: Remaining bile ducts within the pancreas; Group D: Main pancreatic duct.

To clearly evaluate different segments of the biliary tract, we divided patients into three groups according to which ducts were imaged: intrahepatic bile ducts (labeled Group A), extrahepatic bile ducts (Group B), and the remaining areas within the pancreas (Group C). The bile ducts (target) and adjacent liver tissue (reference) were the ROIs for Group A and Group B, whereas the bile ducts within the pancreas (target) and adjacent pancreas tissue (reference) were the ROIs in Group C. The final value was the average of the two measurements. Given that the main pancreatic duct is an independent drainage system anatomically, with distinct imaging characteristics and evaluation priorities compared to the biliary system, it was designated as a separate Group D for assessment. For this group, the ROI comprised the pancreatic duct (target) and adjacent pancreatic tissue (reference) measured at six levels, with the final value being the average of these six measurements. For both Group C and Group D, the “adjacent pancreas tissue” was uniformly defined as homogeneous pancreatic parenchyma immediately surrounding the target duct, free from visible vessels, calcifications, or artifacts (Fig. **1**).

All images were first marked in 40 keV images *via* 3D Slicer (5.6.1), after which markings were copied onto other images. The CT values and Standard Deviations (SDs) of the ROIs were calculated *via* SimpleITK (2.3.1). The image signal intensity (the CT value of the target area), image noise (defined as the Standard Deviation (SD) of the target area), Signal-to-Noise Ratio (SNR), and Contrast-to-Noise Ratio (CNR) served as evaluation indicators. The SNR and CNR were calculated according to the following formulas [11, 14]:

SNR = HU_target_/SD_target_

CNR = | HU_target_ − HU_reference_| /SD_target_

### 2.5 Subjective Image Analysis

The virtual monochromatic images and PEIs were independently reviewed by two experienced radiologists with more than 10 years of expertise in abdominal radiology who were blinded to the specific energy levels of the images assessed.

A 5-point Likert scale was used to rate the ducts’ conspicuity and overall image quality. The visibility of the bile and pancreatic ducts was evaluated and received a minimum score of 1 when the ducts were barely visible and a maximum score of 5 when the ducts were distinctly and confidently visible. The overall image quality scale score also begins at 1, which corresponds to poor image quality due to high image noise and/or indistinct duct visibility, and increases to 5, which indicates optimal image quality with minimal noise perception, no issues with low-contrast resolution, superior clarity of the ducts, and distinct visibility of the surrounding tissues [12].

### 2.6 Statistical Analysis

The distribution of the data was evaluated *via* the Kolmogorov‒Smirnov test. Continuous variables are expressed as medians [Interquartile Ranges (IQRs)]. The equality of variance was assessed using Levene’s test. For statistical comparisons, Friedman’s test was employed, followed by Dunn’s multiple-comparison test as a post hoc analysis with Bonferroni correction to control the false positive rate. Interreader agreement for subjective indicators was evaluated *via* the kappa coefficient (κ), with the following interpretation: κ = 0.81–1.00, excellent; κ = 0.61–0.80, substantial; κ = 0.41–0.60, moderate; κ = 0.21–0.40, fair; and κ = 0.00–0.20, poor. Interobserver agreement was calculated before the consensus reading, which involved 2 raters.

A value of *P* < 0.05 (2-sided) was considered to indicate statistical significance. All the statistical analyses were conducted *via* SPSS version 27.0 and GraphPad Prism version 9. The study followed the STROBE guidelines for observational studies; the completed checklist is provided in Table **S1**.

## 3 RESULTS

### 3.1 Patients

A total of 75 patients were enrolled in this study retrospectively. The cohort included 39 men and 36 women, with a median age of 59 (IQR: 54, 68) years. Among the enrolled patients, 80% had non-pancreaticobiliary cancer as a comorbidity (Table **1**).

### 3.2 Comparison of Subjective Image Analysis

Two radiologists demonstrated substantial agreement across the two subjective ratings. The κ values for duct conspicuity and overall image quality were 0.663 and 0.693, respectively.

**Fig. (2):**
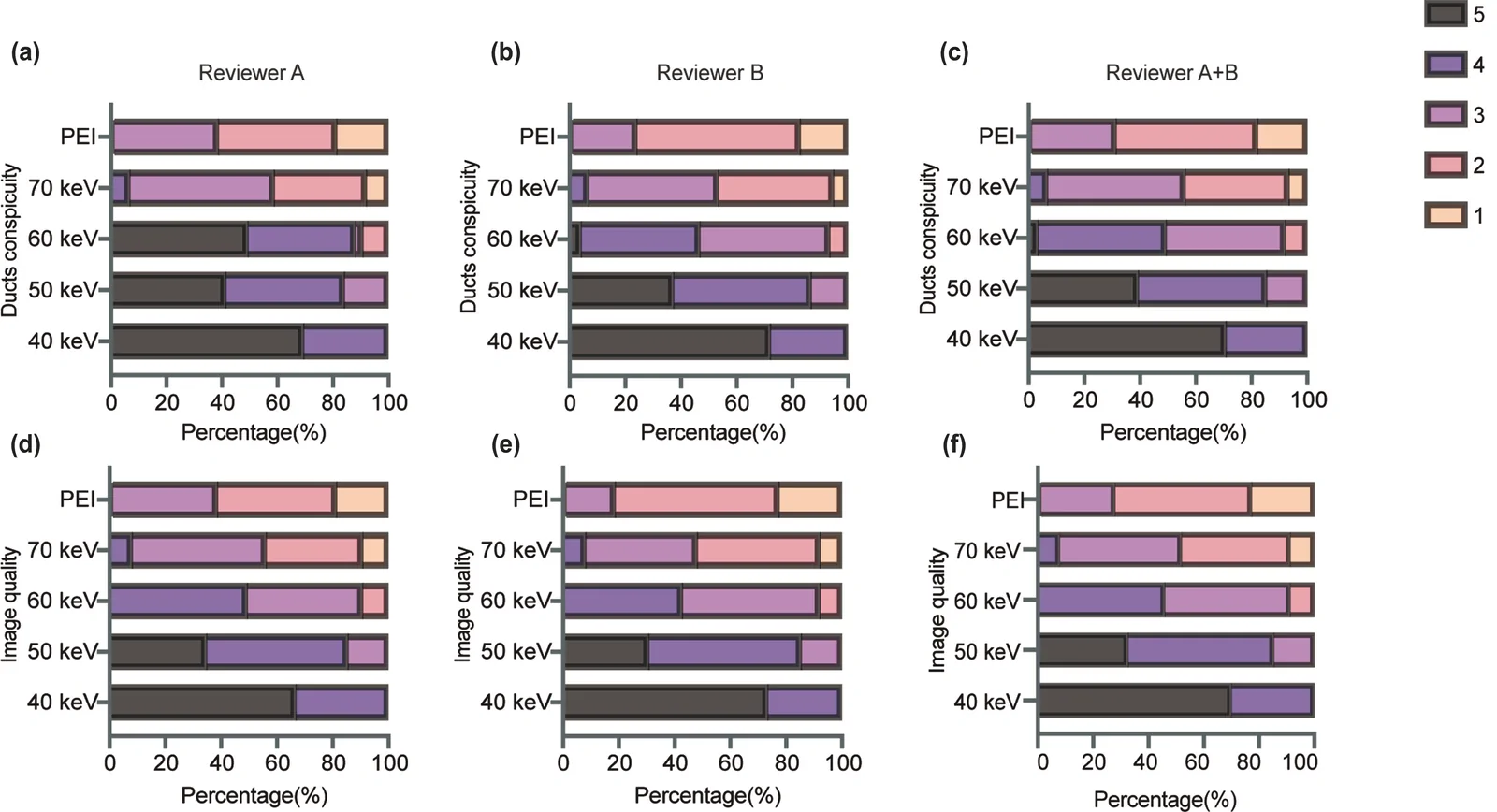
Frequency distribution of subjective image quality scores. Reviewer A (**a** and **d**), Reviewer B (**b** and **e**), and their combined total scores (**c** and **f**).

**Fig. (3):**
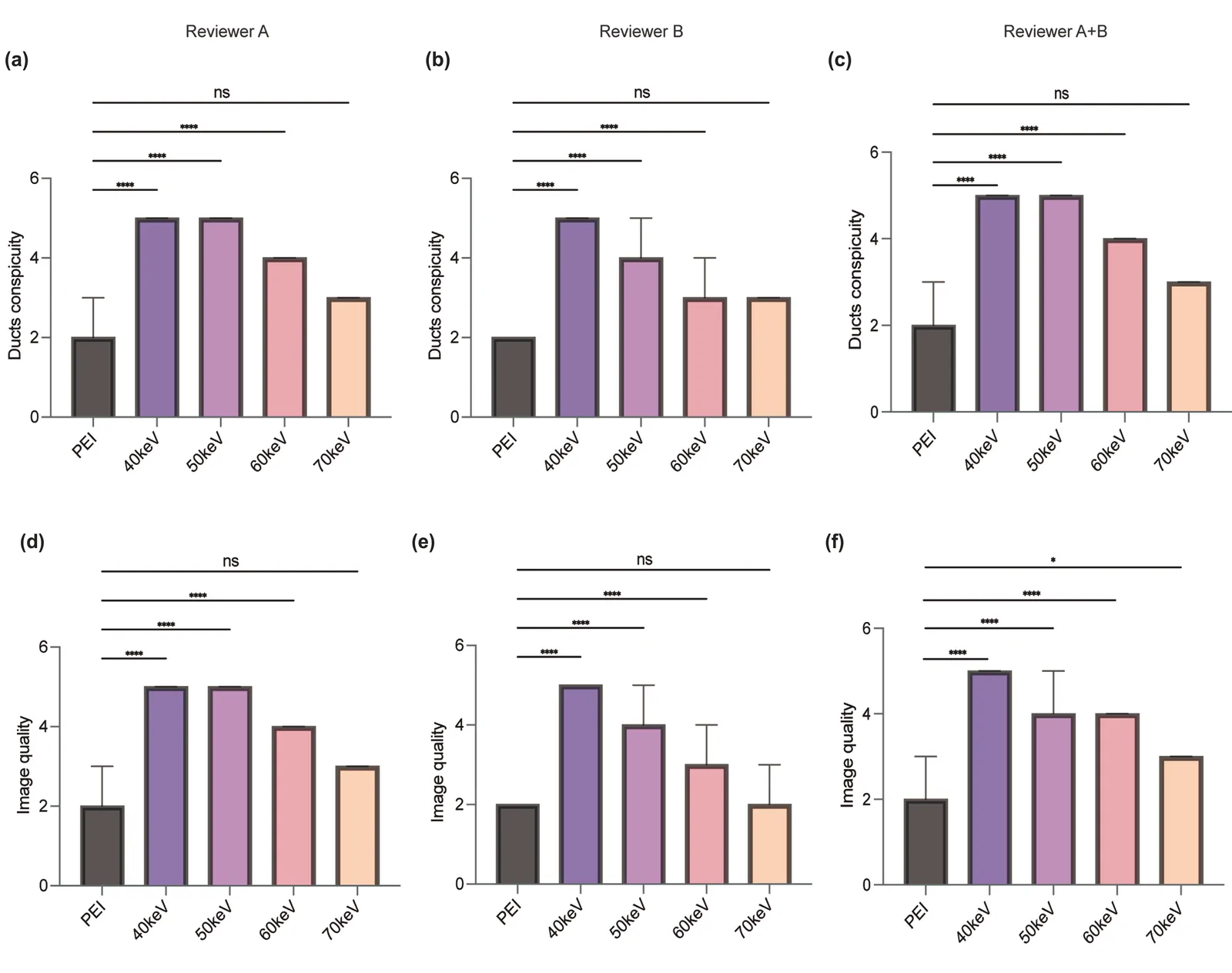
Subjective image quality score. Bar chart displays the results of Reviewer A (**a** and **d**), Reviewer B (**b** and **e**) and their combined total scores (**c** and **f**). ***p<0.001, **p<0.01, *p<0.05, ns: not significant. PEIs, polyenergetic images.

Significant differences were observed between the PEIs and VMIs at 40, 50, and 60 keV in terms of duct conspicuity [PEI: 2 (2, 3), 40 keV: 5 (4, 5), 50 keV: 5 (4, 5), 60 keV: 4 (3, 4), all *p* < 0.00^1^] and overall image quality [PEI: 2 (2, 3), 40 keV: 5 (4, 5), 50 keV: 4 (4, 5), 60 keV: 4 (3, 4), all *p* < 0.00^1^] (Figs. **2** and **3**, Table **2**). A representative case illustrating the improvement in image quality across different energy levels (40 keV, 70 keV, 100 keV), compared with PEIs, is shown in Figure (**S1**).

### 3.3 Comparison of Objective Image Analysis

#### 3.3.1 CT Values

**Fig. (4):**
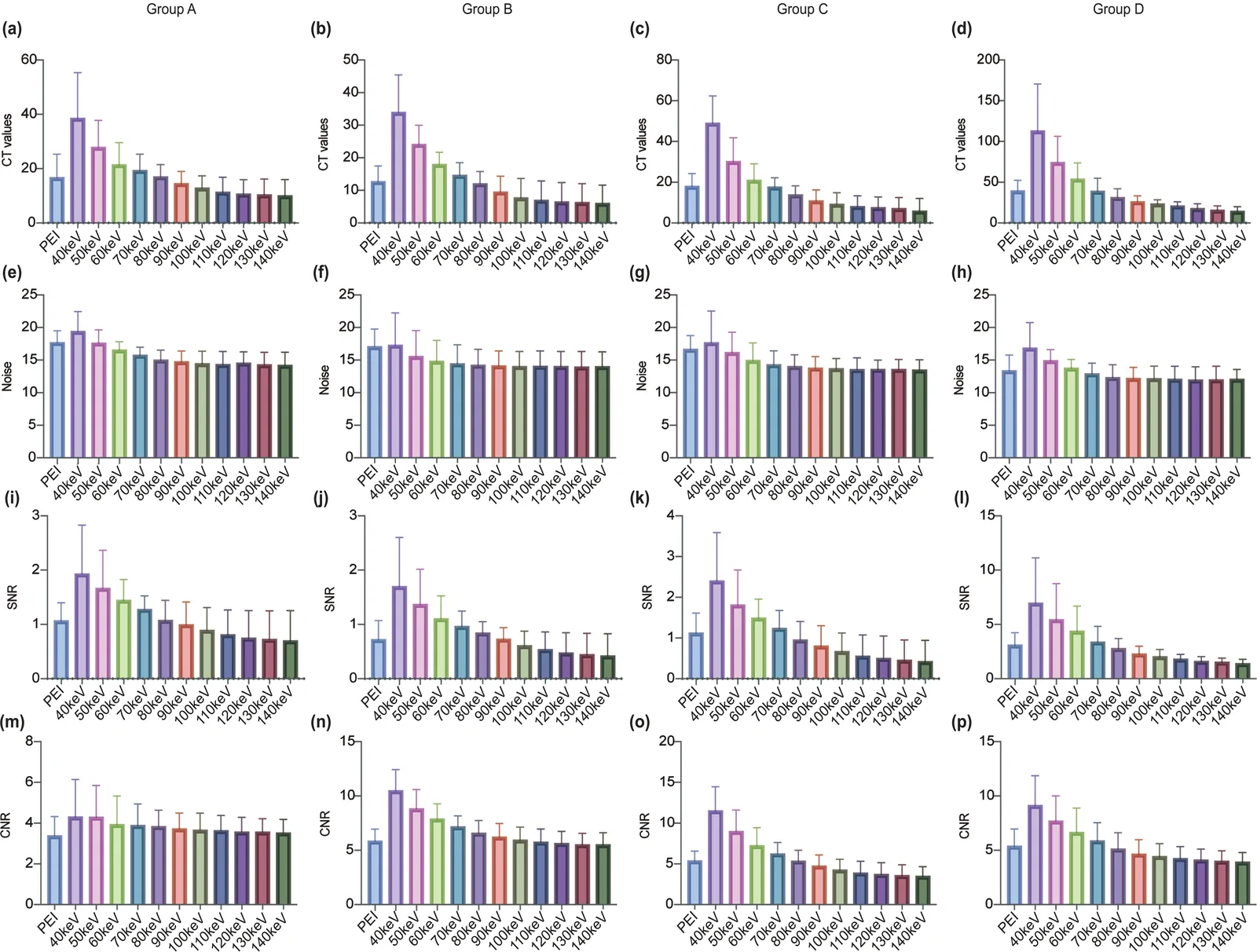
Objective image quality score. Bar charts of the CT values (**a**–**d**), noise (**e**–**h**), SNR (i–l), and CNR (**m**–**p**) of the four groups. CNR, contrast-to-noise ratio. SNR, signal-to-noise ratio. PEIs, polyenergetic images. Group A: Intrahepatic bile ducts; Group B: Extrahepatic bile ducts; Group C: Remaining bile ducts within the pancreas; Group D: Main pancreatic duct.

In all the groups, as the energy level increased from minimal to maximal, a consistent stepwise decrease in the CT value was observed (Fig. **4a**–**d**).

Compared with PEIs, VMIs at 40 keV presented significantly higher CT values across all groups [Group A: 38.49 (24.06, 55.36) *vs.* 16.62 (10.86, 25.34); Group B: 33.87 (24.07, 45.43) *vs.* 12.65 (7.75, 17.49); Group C: 48.88 (32.75, 62.38) *vs.* 17.88 (12.18, 24.25); Group D: 112.72 (74.23, 170.51) *vs.* 38.94 (27.11, 52.17); all *p* < 0.00^1^] (Table **3**).

#### 3.3.2 Noise

For all the groups, the image noise values of the VMIs at different energy levels decreased in a stepwise manner from 40 to 140 keV (Fig. **4e**–**h**).

In Group A, Group B and Group C, the image noise values were not significantly different between the VMIs at 40 keV [Group A: 19.38 (16.34, 22.44) *vs.* 17.65 (15.32, 19.52), *p* = 0.499; Group B: 17.27 (15.35, 22.23) *vs.* 17.04 (15.38, 19.76), *p* > 0.99; Group C: 17.64 (15.63, 22.54) *vs.* 16.64 (15.38, 18.76), *p* > 0.99] and the PEIs. However, in Group D, compared with the PEIs, only the VMIs at 40 keV presented significantly higher image noise values [16.83 (11.83, 20.77) *vs.* 13.34 (10.41, 15.78), *p* = 0.00^7^].

#### 3.3.3 SNR

The Signal-to-Noise Ratios (SNRs) of the VMIs decreased in a stepwise manner from 40 to 140 keV across all four groups (Fig. **4i**–**l**).

Quantitative analysis confirmed a significantly greater CNR for 40 keV VMIs than for PEIs across all groups [Group A: 1.93 (1.25, 2.83) *vs.* 1.06 (0.65, 1.40); Group B: 1.69 (1.17, 2.60) *vs.* 0.72 (0.44, 1.07); Group C: 2.40 (1.77, 3.59) *vs.* 1.12 (0.70, 1.61); Group D: 6.94 (4.06, 11.13) *vs.* 3.08 (1.95, 4.24); all *p* < 0.00^1^].

#### 3.3.4 CNR

Similarly, the CNR of the VMIs decreased in a stepwise manner from 40 to 140 keV across all four groups (Fig. **4m**–**p**).

Compared with that of PEIs, the CNR of 40 keV VMIs significantly improved across all groups [Group A: 4.30 (2.79, 6.15) *vs.* 3.37 (2.52, 4.32), *p*=0.013; Group B: 10.46 (8.41, 12.43) *vs.* 5.83 (4.47, 6.94), *p* < 0.001; Group C: 11.50 (9.05, 14.48) *vs.* 5.34 (4.31, 6.56), *p* < 0.001; Group D: 9.11 (7.00,11.86) *vs.* 5.36 (4.34, 6.95), *p* < 0.00^1^].

## 4 DISCUSSION

This study represents a systematic evaluation that incorporates normal biliary and pancreatic ducts to assess the impact of DSCT-generated VMIs across various energy levels (40–140 keV). The demonstration of significant visualization improvements in normal, nondilated ducts suggests even greater diagnostic potential for pathological dilated conditions. Our findings revealed a consistent, stepwise reduction in CT values, image noise, the CNR, and the SNR as the energy level increased from 40 keV to 140 keV. Notably, within the evaluated spectrum, the lowest-energy VMI at 40 keV demonstrated optimal CNR and SNR values for duct visualization.

Current imaging modalities for biliary-pancreatic evaluation each have limitations. Ultrasound offers high specificity but variable sensitivity due to gas interference [15]. Conventional CT provides mixed-energy imaging with limited tissue differentiation and potential artifact interference. MRI demonstrates high accuracy but is associated with higher costs and accessibility barriers. While ERCP allows detailed ductal assessment, its invasive nature leads to lower patient acceptance and results in notable complications (0.5–5%), including pancreatitis, bleeding, and perforation [2, 16]. Recently, DSCT has emerged as a novel and promising imaging modality for radiological evaluation, as it combines the advantages of MRI and conventional CT. Moreover, DSCT is distinctive because of its perfectly matched dual-measurement projection data in temporal and spatial coregistration and for its ability to conduct energy spectrum scanning simultaneously with conventional scanning without any additional steps or radiation agents [17]. Additionally, spectral CT can provide monochromatic images between 40 and 140 keV, allowing physicians to select the most ideal monochromatic image on the basis of clinical needs, that is, to achieve the best CNR.

The results of our study revealed that across all the groups, as the energy level increased from 40 to 140 keV, the CT value, SNR, and CNR decreased accordingly, and low VMIs, especially 40 keV VMIs, were significantly superior to PEIs. These findings are consistent with previously published research. Using DSCT, Nagayama *et al.* [18] revealed that when the pancreas was imaged, the CNR peaked at 40 keV and gradually decreased from 40 keV to 100 keV. They reached a comparable conclusion in their study on hypovascular hepatic metastases. Liu *et al.* [3] reported that the three indices were inversely correlated with VMIs from 40 to 70 keV and with those of 40 keV virtual monoenergetic images, which provide the best subjective and objective image quality for gastric cancer. The superior image quality at 40 keV is primarily attributed to the photoelectric effect, which dominates X-ray absorption at lower energies, especially near the k-edge of iodine (33.2 keV). At this energy level, iodine within the contrast-filled ducts exhibits a dramatically higher mass attenuation coefficient compared to surrounding soft tissues, thereby maximizing object contrast. These findings suggest that within 40–140 keV, low energy levels, particularly 40 keV, may be optimal for soft tissue imaging. This study explored the impact of different energy levels on DSCT image quality for imaging of the bile and pancreatic ducts. Unlike previous studies that have investigated the diagnostic value of DSCT for specific diseases, this study exclusively included patients without pancreaticobiliary duct abnormalities. This decision was based on established evidence that conventional CT provides adequate visualization of dilated ducts but performs poorly for nondilated ducts and results in significantly reduced detection rates. Moon *et al.* reported substantially reduced CT sensitivity for the detection of choledocholithiasis in the setting of biliary dilatation (72.7% *vs.* 88.9% for bile duct diameters > 10 mm *versus* ≤ 10 mm, respectively) [19]. Similarly, Ko *et al.* reported significantly lower direct detection rates of pancreatic carcinoma by CT in patients without pancreatic duct dilation than in those with dilated ducts [20]. By focusing on patients with normal ductal anatomy-since overt diseases that cause ductal dilation would otherwise diminish the detection challenge on conventional CT and mask the potential advantages of DSCT-we aimed to more precisely evaluate the ability of DSCT to improve the visualization of subtle ductal structures. Notably, our study revealed that DSCT significantly enhances the visualization of bile and pancreatic ducts that have not been dilated because of disease, even when the ducts are very narrow.

Image noise is an important factor in assessing image quality. In our study, we found that the image noise value is negatively correlated with the energy level. However, the image noise in VMI reconstructions at 40–60 keV is almost the same as that in polychromatic images. This apparent discrepancy-where noise did not increase as theoretically expected at the lowest energies-may be explained by the advanced noise-suppression algorithms integrated into the vendor-specific spectral reconstruction pipeline, which are designed to compensate for increased quantum noise at low keV levels.

A detailed group-wise analysis further revealed a distinct pattern. In Group A, B, and C, the image noise values showed no significant difference between 40 keV VMIs and PEIs [Group A: 19.38 (16.34, 22.44) *vs.* 17.65 (15.32, 19.52), *p* = 0.499; Group B: 17.27 (15.35, 22.23) *vs.* 17.04 (15.38, 19.76), *p* > 0.99; Group C: 17.64 (15.63, 22.54) *vs.* 16.64 (15.38, 18.76), *p* > 0.99]. In contrast, in Group D (main pancreatic duct), the 40 keV VMIs presented significantly higher noise compared to PEIs [16.83 (11.83, 20.77) *vs.* 13.34 (10.41, 15.78), *p* = 0.00^7^]. The unique noise behavior in Group D may be attributed to both methodological and anatomical considerations: 1) Methodologically, Group D employed an average of six measurements along the duct course to comprehensively assess its tortuous anatomy, whereas Groups A–C used averages from two measurements. This approach, while more representative, may integrate a wider range of background noise variations; 2) Anatomically, the main pancreatic duct is embedded within the relatively homogeneous pancreatic parenchyma. At low keV (40 keV), the inherent pixel-intensity fluctuation within such uniform soft tissue becomes more pronounced. Compared to the mixed-tissue environments surrounding the bile ducts in Groups A–B (*e.g.*, liver, fat), the homogeneous pancreatic background may allow this inherent soft-tissue noise to be more prominently captured and reflected in the averaged measurement. Therefore, the elevated noise in Group D does not indicate inferior image quality of 40 keV VMIs for pancreatic duct visualization. Instead, it reflects how inherent background noise manifests under a specific measurement strategy and anatomical context. This underscores the importance of considering anatomy-specific and methodology-aware interpretations when optimizing and evaluating spectral CT protocols.

Moreover, previous studies have shown the same results: as the monoenergetic keV decreases, the image noise increases [21, 22]. We believe that although overall image noise in low-keV images is significantly more pronounced, the increased SNR and CNR can be used to increase radiologists’ confidence in the qualitative assessment of image quality, provided that the image noise is within an acceptable level.

However, several limitations remain. First, we explicitly acknowledge that this study was retrospective and conducted at a single tertiary center with a relatively modest sample size, factors that may have introduced selection bias and limited the statistical power and generalizability of the findings. Consequently, the reproducibility and external validity of our findings may be constrained. A prospective, multicenter study with a larger, more diverse cohort is warranted to validate and strengthen our conclusions for broader clinical adoption. Second, the energy level analysis was restricted to 40–140 keV. Extremely low energies (< 40 keV) or finer intervals (*e.g.*, 5 keV steps around optimal ranges) might reveal additional nuances in image optimization. Thirdly, this study relied solely on a single vendor-specific DSCT system and reconstruction algorithms (iDose^4^, spectral level 4). The use of these vendor-specific algorithms-iDose^4^ for conventional polychromatic images and spectral level 4 for spectral reconstructions-may have systematically influenced baseline noise and contrast calculations, potentially affecting the measured differences between PEI and VMI. Thus, the reported advantages of 40 keV images in terms of CNR and SNR may partly reflect the optimization characteristics of this specific reconstruction pipeline in the low-energy range. This may limit the generalizability of our findings to other spectral CT platforms, as differences in hardware and reconstruction algorithms across manufacturers could influence the optimal keV and the magnitude of image quality improvement. A direct, multi-vendor comparative study would be valuable to assess the transferability of our optimization strategy. Fourthly, the subjective image quality assessment was performed by only two radiologists. While the inter-observer agreement (Kappa) reached a “substantial” level, the inclusion of additional readers or repeated assessments in future studies would further strengthen the reliability and robustness of the subjective evaluation. Finally, approximately 80% of the patients in this study had non-pancreatobiliary malignancies. Although imaging excluded metastasis or direct invasion to the liver, biliary tract, or pancreas, the potential influence of these systemic disease states on the local tissue microenvironment remains a theoretically unmeasurable confounding factor. Prospective studies involving healthy volunteers in the future will help establish a purer imaging baseline.

## CONCLUSION

In conclusion, this study demonstrates that low-keV virtual monoenergetic images, especially at 40 keV, provide superior image quality for visualizing normal biliary and pancreatic ducts within the 40–140 keV range of dual-layer spectral CT. These findings suggest that incorporating low-keV VMI reconstructions into routine clinical protocols could enhance the detection and evaluation of subtle ductal structures, particularly in cases where conventional CT offers limited visualization. Therefore, 40 keV VMIs represent a promising technical refinement for noninvasive pancreatobiliary imaging, with potential to improve diagnostic confidence in both normal and early pathological states.

## Figures and Tables

**Table 1:** Patient characteristics.

**Item**	**Total (n = 75)**
Age, year	59 (54, 68)
Sex	
Male	39 (52.0)
Female	36 (48.0)
Comorbidities	
Digestive disease	
Stomach	
Gastric cancer	15 (20.0)
Gist	1 (1.3)
Ectopic pancreas	1 (1.3)
Esophageal cancer	5 (6.7)
Colorectal cancer	18 (24.0)
Respiratory disease	
lung cancer	11 (14.7)
Pulmonary nodule	1 (1.3)
Reproductive disease	
Endometrial cancer	1 (1.3)
Breast tumors	6 (8.0)
Immunological disorders	
Splenic cancer	1 (1.3)
Urological diseases	
Bladder cancer	3 (4.0)
Other classification	12 (16.0)

**Note:** Data are presented as the median (Interquartile range) or number (%).

**Table 2:** Comparison of subjective observation indices of image quality.

	**PEI**	**Mono40keV**	**Mono50keV**	**Mono60keV**	**Mono70keV**
Duct depiction	2 (2, 3)	5 (4, 5)	5 (4, 5)	4 (3, 4)	3 (2, 3)
Quality	2 (2, 3)	5 (4, 5)	4 (4, 5)	4 (3, 4)	3 (2, 3)

**Note:** PEI, poly energy images. Data are presented as median (Interquartile range).

**Table 3:** Comparison of objective observation indices of image quality.

**Variable**	**GroupA**	**P-value**	**GroupB**	**P-value**	**GroupC**	**P-value**	**GroupD**	**P-value**
CTvalues								
PEI	16.62(10.86, 25.34)		12.65(7.75, 17.49)		17.88(12.18, 24.25)		38.94(27.11, 52.17)	
40keV	38.49(24.06, 55.36)	<0.001	33.87(24.07, 45.43)	<0.001	48.88(32.75, 62.38)	<0.001	112.72(74.23, 170.51)	<0.001
50keV	27.78(17.67, 37.75)	<0.001	24.05(16.28, 29.99)	<0.001	30.05(22.70, 41.74)	<0.001	74.12(49.64, 106.39)	<0.001
60keV	21.35(14.57, 29.56)	0.335	17.91(11.37, 21.69)	0.0467	20.91(15.64, 29.00)	0.449	53.65(37.41, 73.74)	0.257
70keV	19.26(11.58, 25.32)	>0.99	14.58(8.54, 18.48)	>0.99	17.56(11.71, 22.17)	>0.99	38.71(27.44, 54.95)	>0.99
80keV	16.93(9.47, 21.45)	>0.99	11.96(6.54, 15.76)	>0.99	13.71(9.08, 18.25)	0.114	30.87(21.29, 41.88)	0.330
90keV	14.42(8.12, 18.96)	0.452	9.45(5.55, 14.37)	0.238	10.75(6.47, 16.24)	0.0006	25.73(16.15, 33.29)	0.0007
100keV	12.75(7.08, 17.31)	0.075	7.64(5.06, 13.65)	0.020	9.09(4.53, 14.83)	<0.001	23.00(13.37, 28.38)	<0.001
110keV	11.20(6.70, 16.81)	0.015	6.88(4.29, 12.88)	0.002	7.95(2.87, 13.28)	<0.001	20.58(11.64, 25.96)	<0.001
120keV	10.60(5.21, 15.92)	0.004	6.40(3.58, 12.40)	<0.001	7.41(1.58, 12.76)	<0.001	17.35(10.01, 23.57)	<0.001
130keV	10.28(5.03 ,16.18)	0.001	6.25(3.08, 12.07)	<0.001	6.95(0.79, 12.47)	<0.001	15.43(9.19, 20.98)	<0.001
140keV	9.95(5.02, 15.99)	<0.001	5.94(2.85, 11.61)	<0.001	5.77(0.33, 12.06)	<0.001	14.01(8.25, 19.96)	<0.001
Noise								
PEI	17.65(15.32, 19.52)		17.04(15.38, 19.76)		16.64(15.38, 18.76)		13.34(10.41, 15.78)	
40keV	19.38(16.34, 22.44)	0.499	17.27(15.35, 22.23)	>0.99	17.64(15.63, 22.54)	>0.99	16.83(11.83, 20.77)	0.007
50keV	17.59(14.80, 19.63)	>0.99	15.54(13.83, 19.54)	0.200	16.14(13.70, 19.29)	>0.99	14.89(10.81, 16.60)	>0.99
60keV	16.53(14.06, 17.83)	0.361	14.79(13.35, 18.03)	0.002	14.92(12.87, 17.63)	0.184	13.74(10.05, 15.10)	>0.99
70keV	15.71(13.39, 16.98)	0.008	14.41(12.94, 17.35)	<0.001	14.27(12.50, 16.44)	0.006	12.86(9.94, 14.54)	>0.99
80keV	14.98(13.17, 16.53)	<0.001	14.20(12.90, 16.66)	<0.001	14.00(12.15, 15.82)	<0.001	12.31(9.70, 14.28)	0.693
90keV	14.72(13.20, 16.38)	<0.001	14.10(12.82, 16.41)	<0.001	13.75(11.67, 15.53)	<0.001	12.16(9.64, 13.90)	0.435
100keV	14.43(12.88, 16.37)	<0.001	14.00(12.71, 16.32)	<0.001	13.65(11.53, 15.25)	<0.001	12.11(9.55, 14.07)	0.245
110keV	14.30(12.79, 16.33)	<0.001	14.02(12.65, 16.40)	<0.001	13.51(11.52, 15.34)	<0.001	12.03(9.55, 14.05)	0.194
120keV	14.52(12.57, 16.26)	<0.001	13.99(12.68, 16.32)	<0.001	13.56(11.29, 14.99)	<0.001	11.90(9.66, 13.98)	0.152
130keV	14.26(12.51, 16.19)	<0.001	13.91(12.67, 16.33)	<0.001	13.53(11.35, 15.09)	<0.001	11.96(9.29, 14.05)	0.133
140keV	14.18(12.49, 16.22)	<0.001	13.97(12.72, 16.25)	<0.001	13.45(11.54, 15.04)	<0.001	12.03(9.50, 13.57)	0.102
SNR								
PEI	1.06(0.65, 1.40)		0.72(0.44, 1.07)		1.12(0.70, 1.61)		3.08(1.95, 4.24)	
40keV	1.93(1.25, 2.83)	<0.001	1.69(1.17, 2.60)	<0.001	2.40(1.77, 3.59)	<0.001	6.94(4.06, 11.13)	<0.001
50keV	1.66(1.12, 2.37)	<0.001	1.36(0.99, 2.02)	<0.001	1.80(1.37, 2.67)	<0.001	5.41(3.24, 8.76)	0.002
60keV	1.44(0.92, 1.82)	0.033	1.10(0.78, 1.53)	0.002	1.49(1.02, 1.95)	0.0681	4.36(2.65, 6.69)	0.225
70keV	1.27(0.80, 1.53)	>0.99	0.96(0.56, 1.25)	0.405	1.23(0.79, 1.68)	>0.99	3.36(2.20, 4.82)	>0.99
80keV	1.07(0.63, 1.44)	>0.99	0.84(0.45, 1.05)	>0.99	0.95(0.61, 1.40)	>0.99	2.76(1.66, 3.71)	>0.99
90keV	0.99(0.54, 1.41)	>0.99	0.73(0.36, 0.94)	>0.99	0.80(0.45, 1.30)	0.1961	2.26(1.20, 3.00)	0.056
100keV	0.89(0.46, 1.31)	>0.99	0.60(0.33, 0.88)	>0.99	0.67(0.31, 1.12)	0.008	2.01(1.04, 2.69)	0.0007
110keV	0.81(0.40, 1.27)	>0.99	0.53(0.29, 0.86)	0.411	0.55(0.19, 1.08)	<0.001	1.79(0.88, 2.25)	<0.001
120keV	0.74(0.34, 1.25)	0.525	0.47(0.24, 0.34)	0.138	0.49(0.12, 1.05)	<0.001	1.58(0.82, 2.03)	<0.001
130keV	0.72(0.29, 1.25)	0.266	0.44(0.24, 0.84)	0.051	0.45(0.05, 0.95)	<0.001	1.51(0.70, 1.90)	<0.001
140keV	0.69(0.28, 1.25)	0.168	0.42(0.20, 0.83)	0.026	0.41(0.17, 0.94)	<0.001	1.36(0.59, 1.80)	<0.001
CNR								
PEI	3.37(2.52, 4.32)		5.83(4.47, 6.94)		5.34(4.31, 6.56)		5.36(4.34, 6.95)	
40keV	4.30(2.79, 6.15)	0.013	10.46(8.41, 12.43)	<0.001	11.50(9.05, 14.48)	<0.001	9.11(7.00, 11.86)	<0.001
50keV	4.28(2.84, 5.85)	0.026	8.81(7.16, 10.60)	<0.001	8.96(7.57, 11.61)	<0.001	7.68(6.24, 10.01)	<0.001
60keV	3.92(2.92, 5.33)	0.079	7.87(6.34,9.28)	<0.001	7.22(5.88, 9.44)	<0.001	6.63(5.31, 8.89)	0.044
70keV	3.88(2.93, 4.94)	0.230	7.15(5.70, 8.17)	0.013	6.18(4.97, 7.62)	0.362	5.84(4.55, 7.54)	>0.99
80keV	3.82(2.93, 4.64)	0.559	6.56(5.41, 7.74)	>0.99	5.33(4.37, 6.68)	>0.99	5.10(4.04, 6.62)	>0.99
90keV	3.71(2.91, 4.50)	>0.99	6.19(5.18, 7.47)	>0.99	4.71(3.97, 6.09)	>0.99	4.63(3.76, 5.98)	0.478
100keV	3.65(2.93, 4.49)	>0.99	5.91(4.94, 7.13)	>0.99	4.23(3.61, 5.56)	0.082	4.41(3.44, 5.60)	0.039
110keV	3.62(2.89, 4.38)	>0.99	5.73(4.77, 6.95)	>0.99	3.84(3.29, 5.32)	0.003	4.22(3.25, 5.34)	0.003
120keV	3.56(2.87, 4.29)	>0.99	5.61(4.64, 6.75)	>0.99	3.67(3.10, 5.14)	<0.001	4.08(3.07, 5.11)	<0.001
130keV	3.56(2.88, 4.22)	>0.99	5.49(4.52, 6.56)	>0.99	3.55(2.93, 4.89)	<0.001	3.98(2.92, 4.95)	<0.001
140keV	3.50(2.86, 4.18)	>0.99	5.49(4.43, 6.62)	>0.99	3.46(2.85, 4.66)	<0.001	3.90(2.81, 4.79)	<0.001

**Note:** Data are presented as median (Interquartile range). SNR, signal to noise ratio, CNR, contrast to noise ratio. PEI, poly energy images.

## Data Availability

The data and supportive information are available within the article.

## LIST OF ABBREVIATIONS

DSCT: Dual-layer Spectral CT
VMIs: Virtual Monoenergetic Images
PEIs: Polyenergetic Images
CNR: Contrast-to-Noise Ratio
SNR: Signal-to-Noise Ratio
US: Ultrasound
MRCP: Magnetic Resonance Cholangiopancreatography
ERCP: Endoscopic Retrograde Cholangiopancreatography
CT: Computed Tomography
SDs: Standard Deviations
SD: Standard Deviation
IQRs: Interquartile Ranges

## STANDARDS OF REPORTING

STROBE guidelines were followed.

## References

[1] Li B., Zhang L., Zhang Z.Y., Ni J.M., Lu F., Wu W.J., Jiang C. (2015). Differentiation of noncalculous periampullary obstruction: comparison of CT with negative-contrast CT cholangiopancreatography *versus* MRI with MR cholangiopancreatography.. Eur. Radiol..

[2] Zandrino F., Benzi L., Ferretti M., Ferrando R., Reggiani G., Musante F. (2002). Multislice CT cholangiography without biliary contrast agent: technique and initial clinical results in the assessment of patients with biliary obstruction.. Eur. Radiol..

[3] Liu J.J., Liu W., Jin Z.Y., Xue H.D., Wang Y.N., Yu S.H., Chen J., Wang Y., Yu J.C. (2020). Improved visualization of gastric cancer and increased diagnostic performance in lesion depiction and depth identification using monoenergetic reconstructions from a novel dual-layer spectral detector CT.. Acad. Radiol..

[4] Liu W., Xie T., Chen L., Tang W., Zhang Z., Wang Y., Deng W., Xie X., Zhou Z. (2024). Dual-layer spectral detector CT: A noninvasive preoperative tool for predicting histopathological differentiation in pancreatic ductal adenocarcinoma.. Eur. J. Radiol..

[5] Peng W., Wan L., Zhao R., Chen S., Dong S., Li L., Zhang H. (2024). Novel biomarkers based on dual-energy computed tomography for risk stratification of very early distant metastasis in colorectal cancer after surgery.. Quant. Imaging Med. Surg..

[6] Li R., Li J., Wang X., Liang P., Gao J. (2018). Detection of gastric cancer and its histological type based on iodine concentration in spectral CT.. Cancer Imaging.

[7] Langenbach I.L., Wienemann H., Klein K., Scholtz J.E., Pennig L., Langzam E., Pahn G., Holz J.A., Maintz D., Naehle C.P., Langenbach M.C. (2023). Coronary calcium scoring using virtual non-contrast reconstructions on a dual-layer spectral CT system: Feasibility in the clinical practice.. Eur. J. Radiol..

[8] Schmitt N. (2025). Dual-layer spectral CT for iodine mapping and surrogate perfusion evaluation in cerebrovascular disease.. Eur. Radiol..

[9] Rassouli N., Etesami M., Dhanantwari A., Rajiah P. (2017). Detector-based spectral CT with a novel dual-layer technology: principles and applications.. Insights Imaging.

[10] Hu M., Wei W., Zhang J., Wang S., Tong X., Fan Y., Cheng Q., Liu Y., Li J., Liu L. (2024). Impact of virtual monochromatic images of different low-energy levels in dual-energy CT on radiomics models for predicting muscle invasion in bladder cancer.. Abdom. Radiol..

[11] Huang X., Gao S., Ma Y., Lu X., Jia Z., Hou Y. (2020). The optimal monoenergetic spectral image level of coronary computed tomography (CT) angiography on a dual-layer spectral detector CT with half-dose contrast media.. Quant. Imaging Med. Surg..

[12] Doerner J., Hauger M., Hickethier T., Byrtus J., Wybranski C., Hokamp N.G., Maintz D., Haneder S. (2017). Image quality evaluation of dual-layer spectral detector CT of the chest and comparison with conventional CT imaging.. Eur. J. Radiol..

[13] Pan X., Wu Z., Zhao J., Zhang X., Zhang X., Tang L., Yang J., Deng K. (2024). Early rectal neoplasm in dual-layer spectral detector computed tomography: dual-layer spectral computed tomography (CT) images improve tumor detection and staging.. Quant. Imaging Med. Surg..

[14] Zhang C., Zhang Z., Yan Z., Xu L., Yu W., Wang R. (2011). 320-row CT coronary angiography: effect of 100-kV tube voltages on image quality, contrast volume, and radiation dose.. Int. J. Cardiovasc. Imaging.

[15] Gurusamy K.S., Giljaca V., Takwoingi Y., Higgie D., Poropat G., Štimac D., Davidson B.R. (2015). Ultrasound *versus* liver function tests for diagnosis of common bile duct stones.. Cochrane Libr..

[16] Anderson M.A., Fisher L., Jain R., Evans J.A., Appalaneni V., Ben-Menachem T., Cash B.D., Decker G.A., Early D.S., Fanelli R.D., Fisher D.A., Fukami N., Hwang J.H., Ikenberry S.O., Jue T.L., Khan K.M., Krinsky M.L., Malpas P.M., Maple J.T., Sharaf R.N., Shergill A.K., Dominitz J.A., ASGE Standards of Practice Committee (2012). Complications of ERCP.. Gastrointest. Endosc..

[17] Schmidt B., Flohr T. (2020). Principles and applications of dual source CT.. Phys. Med..

[18] Nagayama Y., Tanoue S., Inoue T., Oda S., Nakaura T., Utsunomiya D., Yamashita Y. (2020). Dual-layer spectral CT improves image quality of multiphasic pancreas CT in patients with pancreatic ductal adenocarcinoma.. Eur. Radiol..

[19] Moon J.H., Cho Y.D., Cha S.W., Cheon Y.K., Ahn H.C., Kim Y.S., Kim Y.S., Lee J.S., Lee M.S., Lee H.K., Shim C.S., Kim B.S. (2005). The detection of bile duct stones in suspected biliary pancreatitis: comparison of MRCP, ERCP, and intraductal US.. Am. J. Gastroenterol..

[20] Ko S.E., Choi I.Y., Cha S.H., Yeom S.K., Lee S.H., Chung H.H., Hyun J.J. (2016). Clinical and radiologic characteristics of pancreatic head carcinoma without main pancreatic duct dilatation: using dual-phase contrast-enhanced CT scan.. Clin. Imaging.

[21] Kalisz K., Rassouli N., Dhanantwari A., Jordan D., Rajiah P. (2018). Noise characteristics of virtual monoenergetic images from a novel detector-based spectral CT scanner.. Eur. J. Radiol..

[22] Jia Z., Guo L., Yuan W., Dai J., Lu J., Li Z., Du X., Chen W., Liu X. (2024). Performance of dual-layer spectrum CT virtual monoenergetic images to assess early rectal adenocarcinoma T-stage: comparison with MR.. Insights Imaging.

