# Supplementary material for: Improvement in the Image Quality of Bile and Pancreatic Ducts with Dual-Layer Spectral CT
Source: Curr Med Imaging. 2026 Apr 8;22:E15734056442763. doi: 10.2174/0115734056442763260317072250 (PMC13584084; doi:10.2174/0115734056442763260317072250)
Supplement: Supplementary file 1 [file CMIM-22-E15734056442763_SD1.pdf]

1875-6603/26 Send Orders for Reprints to reprints@benthamscience.net

BENTHAM SCIENCE

Current Medical Imaging

Content list available at: https://benthamscience.com/journals/cmir

Supplementary Material

Improvement in the Image Quality of Bile and Pancreatic Ducts with Dual-Layer Spectral CT

Xiaoyi Bai[1,2,†], Kai Liao[3,†], Zhihan Wu[1,2], Mo Chen[4,5], Wang Rui[1,*] and Kai Deng[1,2,*]

[1]*Department of Gastroenterology and Hepatology, West China Hospital, Sichuan University, Chengdu, China*
[2]*Department of Gastroenterology & Hepatology, Sichuan University-University of Oxford Huaxi Joint Center for Gastrointestinal Cancer, West China Hospital, Sichuan University, Chengdu, China*
[3]*Department of Radiology, West China Hospital, Sichuan University, Chengdu, China*
[4]*Department of General Practice, Hospital of Chengdu Office of People's Government of Tibetan, Chengdu, China*
[5]*Department of General Practice, Tibetan Chengdu Branch Hospital of West China Hospital, Sichuan University, Chengdu, China*

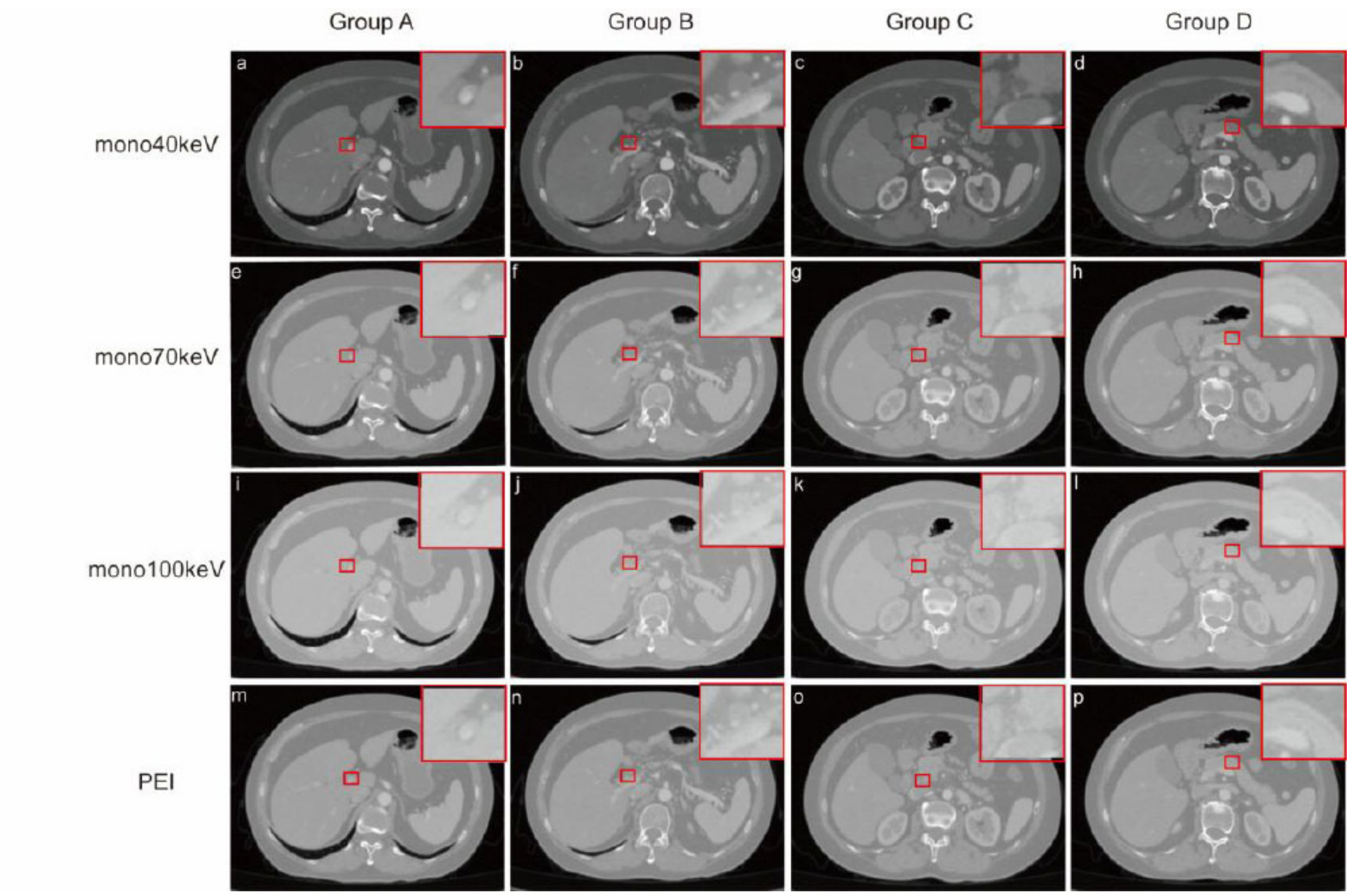

Fig. (S1). Spectral CT images of a representative case showing the biliary and pancreatic at 40 keV (**a–d**), 70 keV(**e–h**) 100 keV and (**i–l**) PEI (**m–p**). Group A: Intrahepatic bile ducts; Group B: Extrahepatic bile ducts; Group C: Remaining bile ducts within the pancreas; Group D: Main pancreatic duct.

DOI: 10.2174/0115734056442763260317072250, 2026, *22*, e15734056442763

Table S1. STROBE Statement—checklist of items that should be included in reports of observational studies.

	Item No.	Recommendation	Page No.	Relevant text from manuscript
**Title and abstract**	1	(*a*) Indicate the study's design with a commonly used term in the title or the abstract	Page1,line 13-14	this retrospective observational
		(*b*) Provide in the abstract an informative and balanced summary of what was done and what was found	Page1,line 16-32	Compared with conventional images, VMIs at low keV exhibit superior image quality; thus, VMIs may be incorporated into routine clinical imaging protocols
**Introduction**				
Background/rationale	2	Explain the scientific background and rationale for the investigation being reported	Page1, line 39-41 and page 2,line 1-26	Biliary and pancreatic duct diseases often evade early detection and present at advanced stages, which leads to profound psychosocial and healthcare system impacts.
Objectives	3	State specific objectives, including any prespecified hypotheses	Page 2,line 27-33	the purpose of this study was to evaluate image quality and determine the optimal energies of VMIs constructed from DSCT ranging from 40 to 140 keV for biliary and pancreatic duct imaging compared with those of PEIs.
**Methods**				
Study design	4	Present key elements of study design early in the paper	Page 3,line 3-8	2. Materials and Methods
Setting	5	Describe the setting, locations, and relevant dates, including periods of recruitment, exposure, follow-up, and data collection	Page 3-4	2. Materials and Methods
Participants	6	a) ***Cohort study***—Give the eligibility criteria, and the sources and methods of selection of participants. Describe methods of follow-up<br>b) ***Case-control study***—Give the eligibility criteria, and the sources and methods of case ascertainment and control selection. Give the rationale for the choice of cases and controls<br>c) ***Cross-sectional study***—Give the eligibility criteria, and the sources and methods of selection of participants	Page 3	2.1. Patients
		d) ***Cohort study***—For matched studies, give matching criteria and number of exposed and unexposed<br>*e)**Case-control study***—For matched studies, give matching criteria and the number of controls per case		
Variables	7	Clearly define all outcomes, exposures, predictors, potential confounders, and effect modifiers. Give diagnostic criteria, if applicable	Page 4	2.4. Objective image analysis<br>and 2.5. Subjective image analysis
Data sources/ measurement	8*	For each variable of interest, give sources of data and details of methods of assessment (measurement). Describe comparability of assessment methods if there is more than one group	Page 4	2.4. Objective image analysis<br>and 2.6. Statistical analysis
Bias	9	Describe any efforts to address potential sources of bias	2.6. Statistical analysis	2.6. Statistical analysis
Study size	10	Explain how the study size was arrived at		
Quantitative variables	11	Explain how quantitative variables were handled in the analyses. If applicable, describe which groupings were chosen and why	Page 4	2.6. Statistical analysis

	Item No.	Recommendation	Page No.	Relevant text from manuscript
Statistical methods	12	(*a*) Describe all statistical methods, including those used to control for confounding	Page 4	2.6. Statistical analysis
		(*b*) Describe any methods used to examine subgroups and interactions		
		(*c*) Explain how missing data were addressed	Page3	2.1. Patients
		(*d*) *Cohort study*—If applicable, explain how loss to follow-up was addressed<br>*Case-control study*—If applicable, explain how matching of cases and controls was addressed<br>*Cross-sectional study*—If applicable, describe analytical methods taking account of sampling strategy		
		(*e*) Describe any sensitivity analyses		
**Results**				
Participants	13*	(a) Report numbers of individuals at each stage of study—eg numbers potentially eligible, examined for eligibility, confirmed eligible, included in the study, completing follow-up, and analysed	Page 4	3.1. Patients
		(b) Give reasons for non-participation at each stage	Page 3	2.1. Patients
		(c) Consider use of a flow diagram		
Descriptive data	14*	(a) Give characteristics of study participants (eg demographic, clinical, social) and information on exposures and potential confounders	Page 4	3.1. Patients
		(b) Indicate number of participants with missing data for each variable of interest		
		(c) *Cohort study*—Summarise follow-up time (eg, average and total amount)		
Outcome data	15*	*Cohort study*—Report numbers of outcome events or summary measures over time		
		Case-control study—Report numbers in each exposure category, or summary measures of exposure		
		Cross-sectional study—Report numbers of outcome events or summary measures	Page 4	3.1. Patients
Main results	16	(*a*) Give unadjusted estimates and, if applicable, confounder-adjusted estimates and their precision (eg, 95% confidence interval). Make clear which confounders were adjusted for and why they were included		
		(*b*) Report category boundaries when continuous variables were categorized		
		(*c*) If relevant, consider translating estimates of relative risk into absolute risk for a meaningful time period		
Other analyses	17	Report other analyses done—eg analyses of subgroups and interactions, and sensitivity analyses		
**Discussion**				
Key results	18	Summarise key results with reference to study objectives	Page 5	
Limitations	19	Discuss limitations of the study, taking into account sources of potential bias or imprecision. Discuss both direction and magnitude of any potential bias	Page 7	
Interpretation	20	Give a cautious overall interpretation of results considering objectives, limitations, multiplicity of analyses, results from similar studies, and other relevant evidence	Page 5-7	
Generalisability	21	Discuss the generalisability (external validity) of the study results	Page 7	A prospective, multicenter study with a larger, more diverse cohort is warranted to validate and strengthen our conclusions for broader clinical adoption.
**Other information**				
Funding	22	Give the source of funding and the role of the funders for the present study and, if applicable, for the original study on which the present article is based	Page 8	Funding

*Give information separately for cases and controls in case-control studies and, if applicable, for exposed and unexposed groups in cohort and cross-sectional studies.

© 2026 The Author(s). Published by Bentham Science Publisher.

This is an open access article distributed under the terms of the Creative Commons Attribution 4.0 International Public License (CC-BY 4.0), a copy of which is available at: https://creativecommons.org/licenses/by/4.0/legalcode. This license permits unrestricted use, distribution, and reproduction in any medium, provided the original author and source are credited.

HOW TO CITE:
Bai X, Liao K, Wu Z, Chen M, Rui W, Deng K. Improvement in the Image Quality of Bile and Pancreatic Ducts with Dual-Layer Spectral CT. Curr Med Imaging, 2026; 22: e15734056442763.
http://dx.doi.org/10.2174/0115734056442763260317072250
